# Analysis of mtDNA sequence variants in colorectal adenomatous polyps

**DOI:** 10.1186/1746-1596-5-66

**Published:** 2010-10-07

**Authors:** Sharifeh Mehrabi, Joyce A Akwe, Gregory Adams, William Grizzle, Xuebiao Yao, Felix O Aikhionbare

**Affiliations:** 1 Department of Medicine, Morehouse School of Medicine, Atlanta, GA, 30310, USA; 2 Department of Pathology and Comprehensive Cancer Center, University of Alabama, Birmingham, AL, 35294, USA; 3 Department of Physiology, Morehouse School of Medicine, Atlanta, GA, 30310, USA

## Abstract

Colorectal tumors mostly arise from sporadic adenomatous polyps. Polyps are defined as a mass of cells that protrudes into the lumen of the colon. Adenomatous polyps are benign neoplasms that, by definition display some characteristics of dysplasia. It has been shown that polyps were benign tumors which may undergo malignant transformation. Adenomatous polyps have been classified into three histologic types; tubular, tubulovillous, and villous with increasing malignant potential. The ability to differentially diagnose these colorectal adenomatous polyps is important for therapeutic intervention. To date, little efforts have been directed to identifying genetic changes involved in adenomatous polyps. This study was designed to examine the relevance of mitochondrial genome alterations in the three adenomatous polyps. Using high resolution restriction endonucleases and PCR-based sequencing, fifty-seven primary fresh frozen tissues of adenomatous polyps (37 tumors and 20 matched surrounding normal tissues) obtained from the southern regional Cooperative Human Tissue Network (CHTN) and Grady Memorial Hospital at Atlanta were screened with three mtDNA regional primer pairs that spanned 5.9 kbp. Results from our data analyses revealed the presence of forty-four variants in some of these mitochondrial genes that the primers spanned; *COX I, II, III, ATP 6, 8, CYT b, ND 5, 6 and tRNAs*. Based on the MITODAT database as a sequence reference, 25 of the 44 (57%) variants observed were unreported. Notably, a heteroplasmic variant C8515G/T in the *MT-ATP 8 *gene and a germline variant 8327delA in the tRNA^lys ^was observed in all the tissue samples of the three adenomatous polyps in comparison to the referenced database sequence. A germline variant G9055A in the *MT-ATP 6 *gene had a frequency of 100% (17/17) in tubular and 57% (13/23) in villous adenomas; no corresponding variant was in tubulovillous adenomas. Furthermore, A9006G variant at *MT-ATP *6 gene was observed at frequency of 57% (13/23) in villous adenomas only. Interestingly, variants A9006G and G9055A were absent in the villous tissue samples that were clinicopathological designated as "polyvillous adenomas". Our current data provide a basis for continued investigation of certain mtDNA variants as predictors of the three adenomatous polyps in a larger number of clinicopathological specimens.

## Findings

Adenomatous polyps are histologically divided into tubular adenomas, villous adenomas, and mixed or tubulo-villous adenomas [1,2]. There is considerable clinicopathological evidence that support adenomatous polyp to be a precursor lesion in most of colorectal cancers. Colorectal malignancy risk correlate with the transformation of a polyp from low risk tubular adenomas to high pure villous adenomas, while the tubulo-villous have an intermediate risk of malignant transformation [2]. At molecular level, changes from tubular to villous through tubulo-villous adenomas may be due to accumulation in mutations of oncogenes, tumor suppressor genes [3-5] and mitochondrial DNA variants within a specific cell and haplogroups [6-8]. These mutations may be adenomatous polyp specific and could potentially be employed as polyp predictors. It is not known, however, whether these mutations are needed to initiate and/or promote tumorigenesis or whether they result from the genomic instability inherent in the resulting colorectal adenocarcinoma [7]. This study examined the possibility of using mitochondrial DNA variants to differentiate the three adenomatous polyps of colon and their matched surrounding paracancerous normal tissues. The mitochondrial genome is particularly susceptible to mutation because of its high local level of reactive oxygen species (ROS) [9,10]. Variants in mtDNA may cause subtle changes in the protein subunits comprising the complexes involved in mitochondrial oxidative phosphorylation (OXPHOS) activity and lead to even higher levels of ROS [11] (ROS are a by product of normal OXPHOS). The accumulation of ROS and oxidative DNA damage during the course of a lifetime may be deleterious and could lead to specific mitochondrial genome markers that may be involved in the development and progression of colorectal cancer [7,12], since colorectal cancer usually occurred in older individuals. In addition, mutant mtDNA may also play a role in tumorigenesis because mutant mitochondria may adapt a replicative advantage, such as deletions/insertions, leading to abnormal cell growth and, ultimately could lead to colorectal adenocarcinoma [13,14].

This study was approved by the Institutional Review Board of Morehouse School of Medicine, the Research Oversite Committee of the Grady Memorial Hospital, Atlanta, Georgia and the University of Alabama at Birmingham School of Medicine, Birmingham, Alabama.

Fifty-seven primary fresh frozen tissues from 3 histologic adenomatous polyps and some of their matched surrounding paracancerous normal tissues (12 tubular and 5 matched surrounding tissues; 11 tubulovillous and 6 matched surrounding tissues; and 14 villous and 9 surrounding tissues; a subset of 10 out of 23 villous adenoma samples were designated as 'polyvillous adenomas' (6 polyvillous and 4 matched surrounding tissues) used in this study were obtained from southern regional Cooperative Human Tissue Network (CHTN) at the University of Alabama, Birmingham, Alabama and the Grady Memorial Hospital, Atlanta, Georgia. Based on the availability of tissue samples not all the 3 adenomatous polyps were matched with surrounding paracancerous normal tissues were analyzed. Colorectal adenomatous polyps were classified as tubular, tubulovillus, and villous based on the defined histologic type, degree of dysplasia, and presence of infiltrating adenocarcinoma in adenoma. Diagnosis was confirmed by histological examinations of biospied specimens for all patients and pathological tumor staging for these was based on American Joint Committee on Cancer. The mean age of the study patients where tissue samples were obtained was 68.3 ± 5.5 years.

To increase the quality of target DNA obtained from small samples, laser-capture microdissection was performed on the tissue samples with the used of an Arctus PixCell II microscope (Arcturus Engineering) for replication of the distance between the polyps and matched surrounding paracancerous normal-appearing cells. MtDNA was extracted from primary frozen tissue samples using centrifugation method according to the manufacturer's protocols (BioVision, Research Products). The extracted mtDNA from 37 adenomatous polyps and 20 matched surrounding paracancerous normal tissues were quantified, and diluted to 50 ng/μl for PCR reaction using three mtDNA regional primer pairs. These primer sets spanned 7392 to 8902 (sense 5'-GGATGCCCCCCACCCTACC-3', antisense 5'-CCTTGTGGTAAGAAGTGGGC-3'; 1530 bp) 8282 to 10088 (sense 5'-CCCCTCTAGAGCCCACTGTAAAGC-3', antisense 5'-GTAGTAAGGCTAGGAGGGTG-3'; 1826 bp) and 13914 to 16527 (sense 5'-CGGATTCTACCCTAGCA-3', antisense 5'-GGAACGTGTGGGCTATTTAGG-3'; 2634 bp) including these mitochondrial genes; *COX I, II, III ATPase 6, 8, CYT b ND 5, 6 and tRNAs *as previously described [6,7]. These resulted in large amplicons that exclude the possibility of amplifying nuclear pseudogenes. MtDNA variants were detected by high resolution restriction digestions and PCR based sequence as previously described [7,12]. Sequences of sense and anti-sense strands were derived with 3100 Genetic Analyzer. Sequences were aligned and compared to human mtDNA [GenBank: J01415] and within samples sequence comparison were made in relationship to the three colorectal adenomatous polyps and the matched normal tissues. For comparison purposes, samples from the general population (n = 50) for which mtDNA was amplified and sequenced using lymphocytes as a surrogates tissues were also used in sequence alignment. MtDNA sequence variants present in both the tumor and matched normal tissue were scored as germ-line variants. Any mtDNA sequence variant that was different between the tumor and the matched normal tissues were scored as somatic mutations. The MITODAT database was used to determine previously published sequences.

Comparison of sequences of mitochondrial DNA obtained from 57 different primary colorectal adenomatous polyps (37 adenomatous polyps cases and 20 matched surrounding paracancerous normal tissues) with the MITODAT database [#J01415] reference revealed 44 sequence variants, in parts of these mitochondrial genes; *ATP 6, 8, CYT b and ND 5, 6 and tRNAs*. Nineteen of 44 (43%) variants were previously reported, 25 of 44 (57%) were unreported variants. We found 4 somatic mutations when adenomatous polyps mtDNA variants were compared with matched surrounding paracancerous normal mtDNA variants. The somatic mutations were observed amongst the matched surrounding paracancerous normal tissues of polyvillous adenomas samples only, a designated subset of villous adenoma. The loci of nucleotide variants amongst colorectal adenomatous polyp samples appear to be consistent. The following deletions 8327delA, 13945delA, 13946delT, 14652delA, 14715delA were detected in tRNA^lys^, *ND 5, 6*, and tRNA^glu^, which were mostly found in tubular adenoma (Table 1). These observed slippage mutations (frameshifts) within the mtDNA coding regions might be a reflection of selective constrains and perhaps account for the progression of colorectal adenomatous polyps to adenocarcinoma. Notably, a heteroplasmic variant C8515G/T in the *MT-ATP 8 *gene and a germline variant 8327delA in the tRNA^lys ^was observed in all the tissue samples of the three adenomatous polyps in comparison to the referenced database sequence. A germline variant G9055A in the *MT-ATP 6 *gene had a frequency of 100% (17/17) in tubular and 57% (13/23) villous adenomas; no corresponding variant was in tubulovillous adenomas.

**Table 1:** Frequency of mtDNA variants among colorectal adenomatous polyps

MT GENES/ REGION	MTDNA VARIANTS	% FREQUENCY ADENOMATOUS POLYPS			NOTES
		Tubular	Tubulovillous	Villous	
MT-ATP6	A9006G	-	-	57 *	R
MT-ATP6	G9055A	100	-	57 *	NR
MT-ATP6	A9093G	100	-		R
MT-ATP6	A8860G	100	100	100	R
MT-ATP6	A9169C	75			NR
MT-ATP8	C8515G/T	100	100	100	R
MT-ATP8	G8697A			43**	R
MT-tRNA	8327 del A	100	100	100	R
MT-ATP6	G8864T			17***	R^a^
MT-ATP6	G8865A			17***	R
MT-ATP6	A8340G			17***	R
MT-ATP6	T8363C			17***	R^b^
MT-ATP 6	G8994A		85		NR
MT-ATP6	G8696A		20	43**	NR
MT-ATP6	A8701G		25		R
MT-ND5	C13938G	85			NR
MT-ND5	C13939G	85			NR
MT-ND5	G13940T	85			NR
MT-ND5	C13941A	85			R^c^
MT-ND5	A13942G	85			R
MT-ND5	C13943A	85			R^d^
MT-ND5	13945 delA	75			NR
MT-ND5	13946 delT	85			NR
MT-ND5	C13947G	85			NR
MT-ND5	C13949T	85			NR
MT-ND5	C13960A	70			NR
MT-ND5	A13969C	70			NR
MT-ND5	A13973T	80			NR
MT-ND5	A13974T	80			NR
MT-ND5	A13975G	80			NR
MT-ND5	A13976G	80			NR
MT-ND5	C13977G	80			NR
MT-ND5	C13978G	80			NR
MT-ND5	T13979G	80			NR
MT-ND5	A13995T	80			NR
MT-ND5	G13996T	80			NR
MT-ND5	A14002G			57 *	R
MT-ND5	G14040A			57 *	R
MT-ND5	C14167T	75		57 *	R
MT-ND6	T14180C	70			R
MT-ND6	A14233G			43 **	R
MT-ND6	14652del A			43 **	NR
M-tRNA	14715del A	80		43 **	NR
MT-CYTB	A14782T	90			NR

* Germ-line mutations/polymorphisms found in all our studied villous adenomas tissues samples only, but not in tissue samples designated as polyvillous adenaomas.

**Germ-lines mutations/polymorphisms found in all our studied tissue samples designated as polyvillous adenomas only, but not in villous adenomas tissues samples.

*** Somatic mutations found in matched surrounding paracancerous normal tissues of polyvillous adenomas in our studied tissue samples only.

R Mutations/polymorphisms are previously reported.

NR Mutations/polymorphisms are unreported.

R^a ^Mutation previously reported as G8864A; R^b ^Mutation previously reported as A8363T.

R^c ^Mutation previously reported as C13941G; R^d ^Mutation previously reported as C13943T.

**Figure 1:**
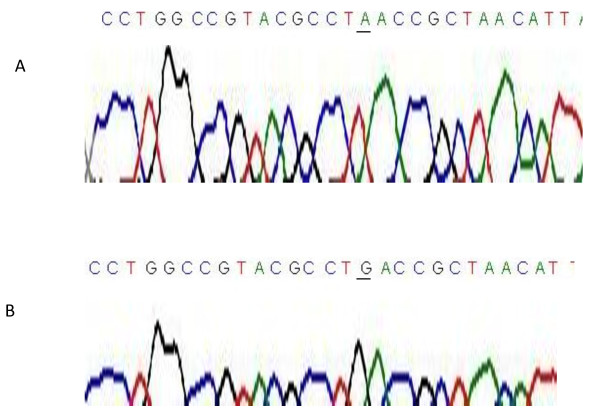
**Chromatograms showing partial *MT-ATP 6 *sequence with at the nucleotide position 9006 germ-line mutation "A → G' (underlined) found in our studied villous adenomas tissues only (B) and with no correspondence mutation in tubular, tubulovillous and the designated polyvillous adenomas sample tissues (A)**.

Furthermore, A9006G variant at *MT-ATP *6 gene was also observed at frequency of 57% (13/23) in villous adenomas only (Table 1, Figure 1). Interestingly, variants A9006G and G9055A were absent in the villous tissue samples that were clinicopathological designated as "polyvillous adenomas". Past study suggested that variants in the mtDNA *ATP synthase subunit 6 *gene seems to contribute positively to the promotion of cancer by prevention of apoptosis [15,16]. A high frequency of 70-85% variants were observed in *ND subunits 5, 6 *and *CYT B *genes (Table 1). The disparate mutational spectra found among the three colorectal adenomatous polyps and the matched surrounding paracancerous normal tissues may reflect difference in underlying colorectal tumor biology as the disease progress as previously described in paragangliomas tumor [17].

Moreover, variants A14633G and 14652delA in the *MT*-*ND6 *gene was found only in the polyvillous adenomas, a designated subset of villous adenomas (Table 1). The difference in mtDNA variants in the *ND gene *between villous and the polyvillous adenomas is particularly striking because it may indicate that polyvillous adenomas is a polyp of invasive carcinoma with high amount of reactive oxygen species. A recent study based on mouse tumor cell line with the application of cytoplasmic hybrid techniques suggested that the main effect of *MT-ND 6 *gene mutations were the defect in the respiratory complex I activity that lead to overproduction of reactive oxygen species. Additionally, this study also reported that mutations in the ND 6 region affected the metastatic potential of the tumor cells and had no effect on the cancer development [15]. Based on the observed mtDNA variants between the villous adenoma and the polyvillous adenoma in this study, the polyvillous adenomas may be considered to be an invasive villous adenocarcinoma. Given that most of the villous adenoma in our samples were large endoscopically unresectable tumors that went to surgical resection even if they were not diagnosed as carcinoma on biopsy as previously described [18]. A study has described two similar colon tumors with the designation of invasive papillary adenocarcinoma and suggested that these tumors may have a more aggressive course than typical colonic adenocarcinoma [19]. Interestingly, Loy and Kaplan [18] used the presence of the epithelial island in desmoplastic stroma to histological distinguish cases of villous adenocarcinoma and villous/tubulovillous adenomas and emphasize the tumor's malignant of villous adenocarcinomas and it's morphologic similarities to villous adenomas.

In this study, we have observed that, in regard to mtDNA from the 3 colorectal adenomatous polyps, there is an appreciable difference in the frequency of sequence variants that potentially distinguished the tubular, tubulovillous and villous adenomas. Also, these finding shows that there are difference in mtDNA variants between villous adenomas and the designated polyvillous adenomas, and mitochondria may be involved in the process of colorectal adenomatous polyps. Based on this study and others, we suggest that classifying polyvillous adenomas as distinct subset of villous adenomas will allow further study of the diagnostically challenging colorectal adenomatous polyps. Perhaps, the designated polyvillous adenomas may be termed as villous adenocarcinoma to emphasize this tumor's malignant potential, morphologic similarities to villous adenomas [18] with genetic differences. However, whether these mtDNA variants identified influence the functionality of the mitochondrial in the colorectal cells during malignancy of the villous adenocarcinoma is a matter that needs to be investigated. Larger, population-based studies are required to quantify the functional role of mtDNA sequence variants in histological colorectal adenomatous polyps and that may subsequently enhance classification of these early colorectal tumors and may facilitate the development of diagnostic adjuncts and an appropriate therapeutic choice. Since we sequenced only a 5.9 kb fragment of the 16.5 kb mitochondrial genome, the number of mtDNA sequence variants that could correlate with the 3 colorectal adenomatous polyps may exceed the number we observed.

## List of abbreviations

AJCC: American Joint Committee on Cancer; ATPASE: ATP synthase 8; COX: Cytochrome B Oxidase; ND: NADH dehydrogenase; MTDNA: Mitochondrial DNA; MITOMAP: Mitochondria databank; OXPHOS: Oxidative phosphorylation; PCR: Polymerase Chain Reaction; ROS: Reactive Oxygen Species; CHTN: Southern Regional Cooperative Human Tissue Network; UAB: University of Alabama-Birmingham.

## Competing interests

The authors declare that they have no competing interests.

## Authors' contributions

SM participated in acquisition of data and drafting the manuscript. JAA helped to draft the manuscript and participated in its review. GAJ participated in the review of the manuscript. WG provided the clinical samples and participated in the review of the manuscript. XY participated in the review of the manuscript. All authors read and approved the final version. FOA conceived, designed and coordinated the study and participated in data analysis and drafted the manuscript.
